# Oxygen Concentration Controls Epigenetic Effects in Models of Familial Paraganglioma

**DOI:** 10.1371/journal.pone.0127471

**Published:** 2015-05-18

**Authors:** Yeng F. Her, Molly Nelson-Holte, Louis James Maher

**Affiliations:** 1 Department of Biochemistry and Molecular Biology, Mayo Clinic College of Medicine, 200 First St. SW, Rochester, MN, 55905, United States of America; 2 Mayo Graduate School, Mayo Medical School and the Mayo Clinic Medical Scientist Training Program, 200 First St. SW, Rochester, MN, 55905, United States of America; Uppsala University, SWEDEN

## Abstract

Familial paraganglioma (PGL) is a rare neuroendocrine cancer associated with defects in the genes encoding the subunits of succinate dehydrogenase (SDH), a tricarboxylic acid (TCA) cycle enzyme. For unknown reasons, a higher prevalence of PGL has been reported for humans living at higher altitude, with increased disease aggressiveness and morbidity. In this study, we evaluate the effects of oxygen on epigenetic changes due to succinate accumulation in three SDH loss cell culture models. We test the hypothesis that the mechanism of α-ketoglutarate (α-KG)-dependent dioxygenase enzymes explains the inhibitory synergy of hypoxia and succinate accumulation. We confirm that SDH loss leads to profound succinate accumulation. We further show that hypoxia and succinate accumulation synergistically inhibit α-KG-dependent dioxygenases leading to increased stabilization of transcription factor HIF1α, HIF2α, and hypermethylation of histones and DNA. Increasing oxygen suppresses succinate inhibition of α-KG-dependent dioxygenases. This result provides a possible explanation for the association between hypoxia and PGL, and suggests hyperoxia as a potential novel therapy.

## Introduction

The SDH complex is a TCA cycle enzyme composed of four highly conserved nuclear-encoded subunits (SDHA-D) localized to the inner mitochondrial membrane. The SDHA and SDHB subunits protrude into the mitochondrial matrix, anchored to the inner mitochondrial membrane by the SDHC and SDHD subunits. SDHA catalyzes the oxidation of succinate to fumarate, and the SDHB subunit contains iron-sulfur clusters that guide the flow of electrons from succinate to ubiquinone in the electron transport chain. Mutations in the genes encoding SDH subunits and SDH assembly factor 2, required for flavination of SDH, predispose carriers to develop PGL in an autosomal dominant fashion [1–6]. Cells in carriers heterozygous for germline SDH defects presumably suffer loss of heterozygosity through a second SDH mutation, leading to tumorigenesis through an unknown mechanism. The succinate accumulation hypothesis proposes that this mechanism involves some combination of pseudohypoxia [7–9], and hypermethylation of histones [10], and DNA [11,12]. Neoplasms associated with mutations in SDH genes include neuroendocrine PGLs and pheochromocytomas (PGLs of the adrenal gland), as well as gastrointestinal stromal tumors, thyroid tumors, and renal cell carcinomas [13].

In normal cells under normoxia, HIF1α and HIF2α are post-translationally hydroxylated by prolyl hydroxylases (PHD) and degraded (S1A Fig), histone demethylation is catalyzed by Jumonji domain histone demethylases (JMHD) (S1B Fig), and 5-methylcytosine (5mdC) residues in genomic DNA are converted to 5-hydroxymethylcytosine (5hmdC) by Ten-eleven-translocation (TET) DNA hydroxylases (S1C Fig), presumably as an intermediate in the DNA demethylation process. All of these enzymes are Fe (II) dioxygenases that bind molecular oxygen, α-KG, and macromolecular substrate at their active sites, catalyzing the oxygenation of the macromolecular substrate with production of succinate and CO_2_ as byproducts. According to the succinate accumulation hypothesis [9], SDH loss causes succinate to accumulate in mitochondria. Succinate diffuses into the cytoplasm and inhibits α-KG-dependent dioxygenases by competing with α-KG at the active site, leading to stabilization of HIFα subunits [9,14] and hypermethylation of histones and DNA [10–12]. The succinate accumulation hypothesis has been supported by recent work using an siRNA strategy to knock down SDHD [9,14] and SDHA/B [11] in HEK293 cells, or using a Cre-lox strategy to produce conditional knockout *Sdhb* mouse chromaffin cells [12] under normoxia.

It has also been suggested that HIFα stabilization is important in human SDH-loss tumorigenesis. Succinate has been shown to be elevated when SDH is lost [15,16]. HIF1α was found to be more prominent in the nuclei of some SDH-mutant pheochromocytomas and PGLs [8]. HIF2α was also overexpressed in some tumors with SDH mutations [17–20], and a causative role of pseudohypoxia in tumor formation was suggested [21].

Because of the involvement of dioxygenase inhibition in PGL tumorigenesis [9–12,14,22], we have been intrigued by the peculiar association between residence at high altitude and increased PGL prevalence and morbidity. The prevalence of skull base and neck PGL is reportedly ten-fold higher in patients residing at high altitude than at sea level [23]. In bovines, the prevalence of carotid body hyperplasia increases to ~40% of animals at high altitude [24]. There is also a positive correlation between higher altitude and phenotypic severity in PGL patients with SDHD defects [25]. Chronic hypoxia exacerbated disease aggressiveness in an asymptomatic PGL patient [26]. There is also striking anecdotal evidence for increased PGL incidence and morbidity in chronically hypoxic patients suffering from respiratory or circulatory disorders [27–30] (William Young, personal communication). To address mechanistically this intriguing synergy between hypoxia and PGL, we created and characterized under different oxygen conditions three SDH loss models of familial PGL. We tested the hypothesis that succinate accumulation in SDH-loss PGL models inhibits PHD, JMHD, and TET dioxygenases as a function of oxygen concentration. We report that cells with SDHB knockdown or *Sdhc* knockout indeed dramatically accumulate succinate, but inhibition of PHD, JMHD, and TET dioxygenases is only observed as oxygen is reduced from ambient conditions to the lower levels expected in tissues. These results support the succinate accumulation hypothesis of PGL tumorigenesis and demonstrate that increasing oxygen suppresses succinate inhibition of α-KG-dependent dioxygenases.

## Materials and Methods

### Institutional approvals

Approvals were received from IRB and IACUC, Mayo Clinic. An animal protocol for this study was reviewed and approved by the Mayo Clinic institutional animal care and use committee. For human-derived material written informed consent from the donor was obtained for the use of samples in research, per the Mayo Clinic IRB.

### Generation and culture of SDHB knockdown cells

Stable lentiviral SDHB knockdown in HEK293 cells was performed as previously described [31,32]. All experiments were at 21% O_2_ unless otherwise specified. Cultures were maintained in DMEM (Gibco) containing 10% FBS and 1% penicillin/streptomycin. Culture medium was replaced 6 h after lentiviral transduction. Cells were cultured an additional 2–3 d to confluence before fluorescence-activated cell sorting of the top 10% mCherry positive cells. Sorted cells were cultured in fresh growth medium for 4–5 d to confluence. Cells were passaged and SDHB knockdown confirmed by western blotting. SDHB knockdown cells were split 1:10 and grown in the indicated oxygen condition (21%, 10%, or 2%) for at least 3 d prior to analysis. Oxygen conditions were maintained by appropriate combination of room air, CO_2_ and N_2_.

### Generation of SDHC conditional knockout mice

SDHC gene trap mouse strain C57BL/6N-Sdhc^tm1a(EUCOMM)Wtsi/^Wtsi was obtained from the European Conditional Mouse Mutagenesis Program, Sanger Center, UK. By crossing *Sdhc* floxed (fl) mice with FLP expressing mice, the *Engrailed* polyadenylation site was excised, yielding loxP recombination sites flanking exon 4 (S2 Fig). The following primers were used for PCR genotyping: LJM-4429 (5’-CT_2_AGA_2_CTGATC_4_TGC_3_) and LJM-4430 (5’-CACTGC_3_G_2_CTCATAT_3_C). *Sdhc* (fl/-) mice were created by mating founder *Sdhc* (-) mice with *Sdhc* (fl) mice. *Sdhc* (fl/-) conditional knockout mice on the CRE^ER^TM background were created by breeding *Sdhc* (fl/-) mice with CRE^ER^-TM mice, allowing disruption of the floxed *Sdhc* allele by CRE recombination between the *loxP* sites upon Tamoxifen (TAM) treatment. Genotyping for CRE-recombined *Sdhc* was done using LJM-4429 and LJM-4612 (5’-G_2_CGAGCTCAGAC_2_ATA_2_CT_2_C). An animal protocol for this study was reviewed and approved by the Mayo Clinic institutional animal care and use committee.

### Generation, culture, and immortalization of Sdhc conditional knockout MEFs (iMEFs)

*Sdhc* fl/- Cre- and *Sdhc* fl/- Cre+ iMEFs were generated and cultured as described [33–35]. Briefly, MEFs were generated at embryonic day 13.5 and cultured in DMEM (Gibco) containing 10% FBS and 1% penicillin/streptomycin. MEFs were frozen at passage 2 and used for immortalization of 1x10^5^ cells by transduction using lentiviruses expressing the SV40 large T antigen [36]. 2000 cells were plated and grown in 15-cm dishes for four passages to eliminate non-immortalized cells. iMEF monolayer cultures were treated with 1 μM TAM for at least 7 d, at which time the medium was replaced every 1 d with fresh medium containing 1 μM TAM with passaging as needed. Treated cells were collected for western blot analysis to confirm loss of SDHB and SDHC. Cultures were then split 1:10 into the indicated oxygen condition (21%, 10%, or 2%) without TAM for at least 72 h prior to analysis.

### Generation, culture, and immortalization of Sdhc conditional knockout kidney cells

Kidneys from five-day-old *Sdhc* fl/- Cre- and *Sdhc* fl/- Cre+ mice were obtained using standard sterile technique, washed with PBS, and dissociated with scissors. Dissociated tissue was treated with collagenase solution (0.5 mg/mL; Sigma) and incubated at 37° C for 2.5 h. The supernatant was removed and pelleted by centrifugation at 1,000 rpm for 10 min. The cell pellet was suspended in DMEM containing 10% FBS and 1% penicillin/streptomycin, and cultured until cells reached 70% confluence prior to immortalization and TAM treatment as described above.

### Immunoblotting

Cells grown in 100 mm plates were harvested and lysed with 120 μL RIPA buffer (50 mM Tris, 5 mM EDTA, 150 mM NaCl, 0.1% SDS, 0.5% deoxycholic acid and 1% NP-40) containing 1X proteinase inhibitor cocktail (Roche) and 1X phosphatase inhibitors (Thermo Scientific) and agitated for 20 min on ice in a 4° C room. Cell lysates were subjected to centrifugation at 14,000 rpm in a clinical centrifuge for 15 min at 4° C, and 100 μL of the supernatant was transferred to a 1.7 mL tube and proteins quantified using a BCA kit (Pierce). Twenty μg of total protein was treated with 1X reducing agent (Life Technologies) in 1X LDS buffer (Life Technologies), heated for 10 min at 95° C, loaded onto a 10% bis-Tris polyacrylaminde gel, and electrophoresed at 150 V for 1.5 h. Proteins were transferred to nitrocellulose followed by blocking with 3% milk for 1 h. The filter was washed with 1X TBST buffer and probed with the appropriate primary antibody dilution overnight. Primary antibody and dilution used for human protein was anti-HIF1α (BD Bioscience, 610958, mouse polyclonal, 1:1000,). Primary antibodies used for mouse proteins were anti-SDHC (Santa Cruz Biotechnology, sc-67256, rabbit polyclonal, 1:1000), anti-HIF1α (1:1000, Abgent). Primary antibodies used for both human and mouse proteins were anti-SDHA (Abcam, ab14715, mouse monoclonal, 1:10000), anti-SDHB (Abcam, ab178423, rabbit monoclonal, 1:1000), anti-actin (Sigma, A2066, rabbit polyclonal, 1:500,), anti-H3 (Santa Cruz Biotechnology, sc-10809, rabbit polyclonal, 1:1000), anti-H3K9me2 (Abcam, ab1220, mouse monoclonal, 1:1000) and anti-H3K27me2 (Abcam, ab24684, rabbit polyclonal, 1:15000), and anti-HIF2α (Novus Biologicals, NB100-132, rabbit polyclonal, 1:1000). Horseradish peroxidase-conjugated anti-rabbit/mouse secondary antibody (GE Healthcare, NA934V or NA931V, 1:15000) and an ECL plus kit (Pierce) were used to detect immunoreactive protein.

### Octyl-α-KG treatment

Octyl-α-KG was prepared as described by [14]. Cells were cultured as described above. When cultures reached 50% confluency, octyl-α-KG (dissolved in DMSO) was added to the culture to a final concentration of 250 μM, followed by incubation for 12 h and western blot analysis as described above.

### TCA cycle metabolite analysis

TCA cycle metabolites were determined by the Mayo Clinic Metabolomic Mass Spectrometry Core. Cell pellets were suspended in 100 μL PBS followed by addition of 20 μL internal standard solution containing [^13^C]-labeled analytes. Cells were sonicated for 60 sec prior to addition of 400 μL chilled methanol/acetonitrile solution to precipitate proteins. After drying the supernatant by centrifugal evaporation, the sample was derivatized with ethoxime and then with 1% N-Methyl-N-(t-butyldimethylsilyl)-trifluoroacetamide and 1% t-butyldimethylchlorosilane before analysis on an Agilent 5975C GC/MS instrument under electron impact and single ion monitoring conditions. Concentrations of lactic acid (m/z 261.2), fumaric acid (m/z 287.1), succinic acid (m/z 289.1), oxaloacetic acid (m/z 346.2), ketoglutaric acid (m/z 360.2), malic acid (m/z 419.3), cis aconitic acid (m/z459.3), citric acid (m/z 591.4), isocitric acid (m/z 591.4), and glutamic acid (m/z 432.4) were measured against a 7-point calibration curve after the same derivatization [37]. The concentration of each metabolite was normalized to the number of cells in each sample and to the corresponding metabolite concentration in the control sample.

### SDH activity

SDH activity was determined using the Complex II Enzyme Activity Microplate Assay Kit (Abcam) according to the manufacturer's instructions. Briefly, cells were lysed in detergent, agitated, incubated on ice for 30 min, and collected by centrifugation at 4° C for 20 min at 14,000 rpm in a clinical centrifuge. The supernatant was collected and the protein concentration determined using a BCA kit (Pierce). Protein was diluted to 60 μg/mL using 1X incubation solution. 50 μL of total protein was distributed to each well of an immunocapture plate, allowing the SDH complex to be immobilized for 2 h. Wells were washed twice with wash buffer and 240 μL activity solution was added to each well with chromogenic assay monitored at 600 nm for 1 h on a plate reader.

### Genomic DNA extraction

Genomic DNA was extracted using a kit (Qiagen) according to the manufacturer's instructions, with minor modifications as described [38]. Briefly, 5x10^6^ cells were washed with ice-cold PBS and lysed with C1 buffer. The lysate was subjected to centrifugation at 1,000 rpm for 10 min in a clinical centrifuge at 4° C, and the supernatant was discarded. Pelleted nuclei were resuspended in 2 mL G2 buffer followed by agitation for 30 s to lyse nuclear membranes. Fifty μL RNase A solution (Thermo Scientific, 10 mg/mL) and 100 μL Proteinase K solution (Sigma, 10 mg/mL) were added, with incubation overnight at 55° C. Genomic DNA was then further purified according to the manufacturer's instructions.

### Enzymatic hydrolysis of genomic DNA

Genomic DNA was hydrolyzed as described in [39]. Briefly, purified genomic DNA was hydrolyzed to mononucleosides using a mixture of deoxyribonuclease I (DNase I), micrococcal nuclease (MNase I), snake venom phosphodiesterase (SVPD), and antarctic phosphatase (AP) as follows. A 40 μL reaction contained 3 μg genomic DNA (pre-heated to 95°C and cooled to room temperature), MNase buffer (New England BioLabs) supplemented with 400 mM MgCl_2_, 4 mM ZnCl_2_, 20 U DNase I (New England Biolabs), 2000 U MNase (New England BioLabs), 5 U AP (New England Biolabs) and 0.4 U SVPD (Worthington). Genomic DNA was digested overnight at 37°C prior to LC-MS analysis.

### LC-MS

Ten μL hydrolyzed genomic DNA solution (~0.6 μg) was subjected to reverse phase HPLC analysis at room temperature using a C18 analytical column (Phenomenex-C18 1.0x250 mm) and Agilent series 1100 instrument (Agilent Technologies). The column was eluted with mobile phase A (0.05 M ammonium formate, pH 5.4; Sigma, 17843), and a gradient of methanol (mobile phase B) at a flow rate of 0.05 mL/min. The gradient specifications were: 0 min: 2% B; 18 min: 10% B; 30 min: 25% B; 35 min: 2% B; and 60 min: 2% B. Absorbance was monitored at 277 nm. Mass spectrometric analysis was performed as described [39]. Briefly, the HPLC instrument was connected in-line with a mass spectrometer (MSD-TOF, Agilent Technologies). MS parameters were: capillary 4,000; nebulizer 20 psi; drying gas 7 L/min; gas temperature 325°C; fragmentor 45 V; skimmer 60 V; Oct 1 DC 37.5 V; Oct RF 250 V. The mass spectrometer was set to positive ion mode for nucleoside detection, and data were analyzed using Agilent MassHunter Quantitative Analysis software.

### Tumor specimens

A tissue array was assembled from paraffin-embedded tissue specimens obtained from the Mayo Clinic archives in accordance to a protocol approved by the Mayo Clinic Institutional Review Board. Selection of tumor tissue was done with the assistance of staff endocrinologists and pathologists. Relevant clinicopathological information was also collected for each case.

### Immunohistochemistry

The staining procedure was performed using a Leica Bond III Stainer (Leica,). Slides were subjected exposed to 10 mM sodium citrate buffer, pH 6.0 for 20 min at 37°C. Slides were incubated with the appropriate primary antibody for 15 min, followed by Polymer Refine Detection System (Leica) processing, including hydrogen peroxidase block, secondary antibody polymer, 3,3’ diaminobenzidine and hematoxylin stain. Specimens were then rinsed for 5 min in tap water. Slides were dehydrated in increasing concentrations of ethyl alcohol and xylene prior to permanent coverslipping in xylene-based media. Primary antibodies were as follows: mouse anti-Hif1α (1:400, Novus Biological), mouse anti-HIF2α (Novus Biologicals, NB100-132, rabbit polyclonal, 1:700) mouse anti-H3K9me2 (1:750, Abcam), rabbit anti-H3K27me2 (ab24684, Abcam, 1:500), rabbit anti-5hmC (39769, Active Motif, 1:4000), and mouse anti-SDHB (ab14714, Abcam, 1:1000).

### Statistical analysis

Data (mean ± standard deviation) are presented. Statistical significance by T-test (*P<0.05 and **P<0.01) is indicated.

## Results

### Generation of SDH loss models of PGL

**Fig 1:**
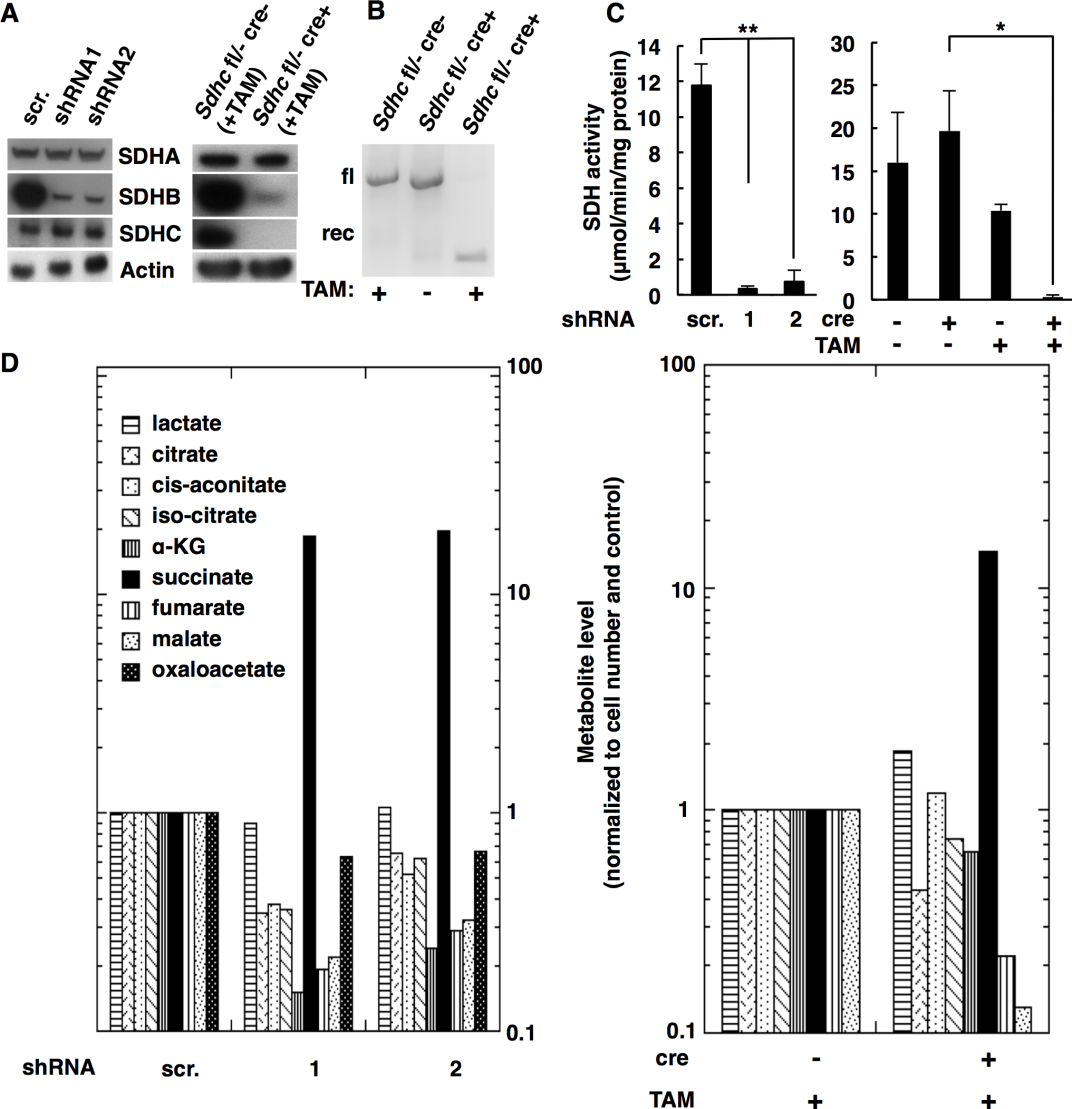
Characterization SDH loss models of PGL: HEK293 SDHB knockdown cells and *SDHC* conditional knockout iMEFs. A. Western blot analysis of HEK293 cells transduced with SDHB silencing lentiviruses (shRNA1 or shRNA2) or control vector (scrambled; scr.) and iMEFs treated with TAM for 7 d. β-actin was used as a loading control. B. PCR genotyping with primers for *SDHC* floxed (fl) allele before and after CRE recombination (rec). C. SDH enzyme assay in lysates from the indicated HEK293 cells and iMEFs (standard deviation reflects triplicates). Data are mean ± standard deviation. Statistical significance by T-test (*P<0.05 and **P<0.01) is indicated. D. Relative metabolite levels in the indicated whole-cell lysates measured by GC/MS analysis. Data are normalized to cell number and the respective control value.

Three SDH loss models of familial PGL were generated for the purpose of evaluating oxygen effects in the context of the succinate accumulation hypothesis. An SDH knockdown model was created by transduction of HEK293 cells with shRNA lentiviral vectors targeting two different sites within the coding region of *SDHB*. *SDHB* was chosen because, inexplicably, defects in *SDHB* have been reported to be associated with a higher malignancy rate [12,40,41] and poorer prognosis than defects in other SDH subunits [42]. Western blot analysis showed that HEK293 cells transduced with shRNA lentiviral vectors (shRNA1 or shRNA2) targeting *SDHB* mRNA resulted in nearly complete depletion of SDHB protein within 12 h (Fig 1A). SDHA and SDHC protein levels were unaffected (Fig 1A). Cell proliferation rates were at least 2-fold slower upon SDHB knockdown relative to cells transduced with the negative control construct (scr). Knockdown cultures showed a small fraction of floating cells with the majority growing as an adherent monolayer. Both fractions were shown to be viable using a Trypan blue exclusion test. We also confirmed loss of SDH enzyme activity by measuring succinate oxidation to fumarate in cell extracts. SDH activity was substantially reduced compared to the control cells (Fig 1C). This result confirms that SDHB knockdown destabilizes the SDH complex, resulting in decreased enzyme activity. This effect is predicted to substantially alter TCA cycle metabolite levels. Indeed, gas chromatography/mass spectrometry (GC/MS) analysis showed that HEK293 cells transduced with shRNA1 or shRNA2 accumulated ~20-fold higher succinate concentrations than negative controls (Fig 1D). Fumarate and malate levels were correspondingly reduced as predicted for SDH blockade. Importantly, α-KG concentrations were also reduced ~8-fold upon SDH knockdown. Thus, the succinate:α-KG ratio was increased by 75-90-fold upon SDH knockdown. It is the dramatic increase in this succinate:α-KG ratio that explains how succinate outcompetes α-KG, inhibiting dioxygenase enzymes.

Additional SDH loss models of PGL were derived from immortalized mouse embryonic fibroblast (iMEF) and immortalized primary kidney (iKidney) cells carrying SDHC gene deletions. We first generated an *Sdhc* conditional knockout mouse as described in Materials and Methods and S3 Fig. Upon treatment with Tamoxifen (TAM), non-functional *Sdhc* alleles are generated in all cells. PCR genotyping of iMEF and iKidney cultures derived from *Sdhc* ^*fl/-*^ cre+ mice treated *in vitro* with TAM showed complete recombinational inactivation of *Sdhc* (Fig 1B and S3A Fig). The doubling rates of cultured *Sdhc*-knockout cells were at least 4-fold lower than the non-recombined control cells. Trypan blue exclusion tests showed the cells to be viable. Western blot analysis demonstrated corresponding complete loss of SDHC protein (Fig 1A, S3B Fig). SDHB was also significantly reduced, consistent with previous reports of SDHB loss when the SDH complex is disrupted [43,44]. Although SDHA protein was stable in the absence of SDHC, SDH activity was completely abolished in these SDH loss models (Fig 1C, S3C Fig). As a result, succinate accumulated 15-fold and 100-fold in *Sdhc* ^*fl/-*^ cre+ iMEFs and *Sdhc* ^*fl/-*^ cre+ iKidney cells, respectively, relative to control cells (Fig 1D, S3D Fig). The succinate:α-KG ratios in *Sdhc* ^*fl/-*^ cre+ iMEFs and *Sdhc* ^*fl/-*^ cre+ iKidney cells were 20-fold and 30-fold higher than in control cells, respectively. Differences in metabolic profiles of iMEFs and iKidney cultures presumably reflect the heterogeneous character of the kidney cells. Interestingly, lactate concentrations were increased at least 10-fold in Sdhc knockout iKidney cells, confirming that disruption of the TCA cycle by loss of SDH complex enforced a glycolytic metabolism on the cells.

### Oxygen concentration controls α-KG-dependent dioxygenase inhibition in SDH loss PGL cell models

**Fig 2:**
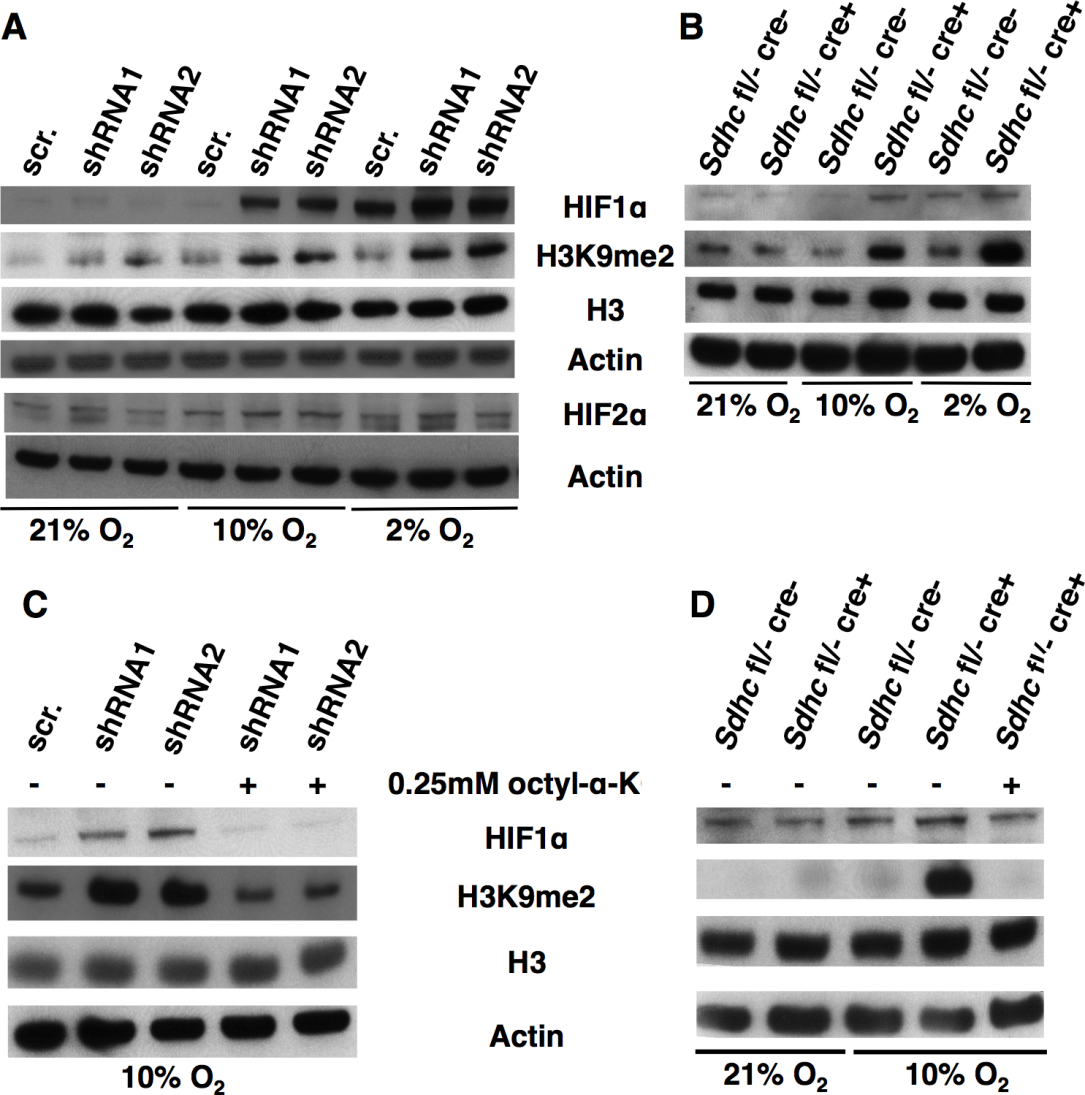
Oxygen concentration-dependence of dioxygenase inhibition. A. HIF1α, HIF2α, and H3K9me2 abundance by western blotting in shRNA1 and shRNA2 cells incubated in 21%, 10%, or 2% oxygen for 72 h. Actin and total H3 serve as loading controls. B. HIF1α, HIF2α, and H3K9me2 abundance by western blotting in SDHC knockout iMEFs incubated in 21%, 10%, or 2% oxygen for 72 h. iMEFs were treated with 1 μM TAM for 7 d prior to analysis. (C-D) Rescue of succinate inhibition of JMHD and PHD inhibition using 0.25 mM octyl-α-ketoglutarate (octyl-α-KG) in SDHB knockdown HEK293 cells and SDHC knockout mouse iMEFs.

It has previously been shown that PHD and JMHD can be inhibited by succinate accumulation in yeast and mammalian cell lines lacking SDH subunits [9–12,14,45]. We first sought to confirm these results with our SDH loss cell culture models of PGL. Surprisingly, western blot analysis showed that neither shRNA knockdown of SDHB in human HEK293 cells, nor SDHC loss in *Sdhc* ^*fl/-*^ cre+ iMEFs or *Sdhc* ^*fl/-*^ cre+ iKidney cells treated with TAM led to accumulation of HIF1α or histone hypermethylation for cells cultured in room air (21% oxygen; Fig 2, S5 Fig). These observations were puzzling because SDH loss cells displayed reduced SDH activity and accumulated at least 10-fold more succinate than controls. We reconsidered the enzyme reaction mechanism of α-KG-dependent dioxygenases. These enzymes operate by an ordered tri-tri reaction mechanism where catalytic rate is proportional to the concentrations of the three reactants, i.e. the macromolecular substrate, α-KG, and molecular oxygen [46,47]. For model dioxygenases, the apparent *K*_m_ for the α-KG substrate is 55 μM [48]. The succinate product of the reaction is also a competitive inhibitor at the enzyme activate site, with an apparent *K*_i_ of 350–460 μM [49]. In the presence of inhibitory succinate, the fraction of uninhibited dioxygenases will depend on succinate concentration. These residual active enzymes may be sufficient to maintain normal levels of HIFαs hydroxylation and histone demethylation if the reaction rate is driven by sufficiently high oxygen concentration (the co-substrate). Because the reaction rate is proportional to oxygen concentration according to the law of mass action, this model suggests that shifting SDH loss cell cultures to lower oxygen concentrations will exacerbate succinate inhibition of dioxygenases. Thus, hypoxia and succinate accumulation should be synergistic in causing dioxygenase inhibition.

To test this hypothesis, we began by studying normal HEK293 cells cultured in the presence of the cell-permeable succinate analog dimethyl succinate (DMS). We measured α-KG-dependent enzyme activity in 21% and 10% oxygen. Consistent with our hypothesis, western blot analysis demonstrated that DMS treatment caused a slightly larger increase in HIF1α and methylated histones in 10% oxygen than in 21% oxygen (S4 Fig). We next examined the effects of oxygen concentration on dioxygenase function in our SDH loss cell culture models of PGL by culturing *Sdhc* ^*fl/-*^ cre+ iMEFs and SDHB knockdown HEK293 cells and controls in 21%, 10%, and 2% oxygen conditions. The 2% oxygen concentration is believed to better reflect physiological conditions [50–53]. Western blot analysis confirmed that reduction of oxygen from 21% to 10% to 2% showed increased HIF1α, HIF2α and histone hypermethylation in experimental cells compared to controls (Fig 2A and 2B and S5 Fig). As expected from the dioxygenase reaction mechanism, HIF1α, HIF2α and H3K9me2 levels increased in control cells upon decreasing oxygen in the absence of succinate accumulation (Fig 2A and 2B). Succinate accumulation further enhanced this effect.

**Fig 3:**
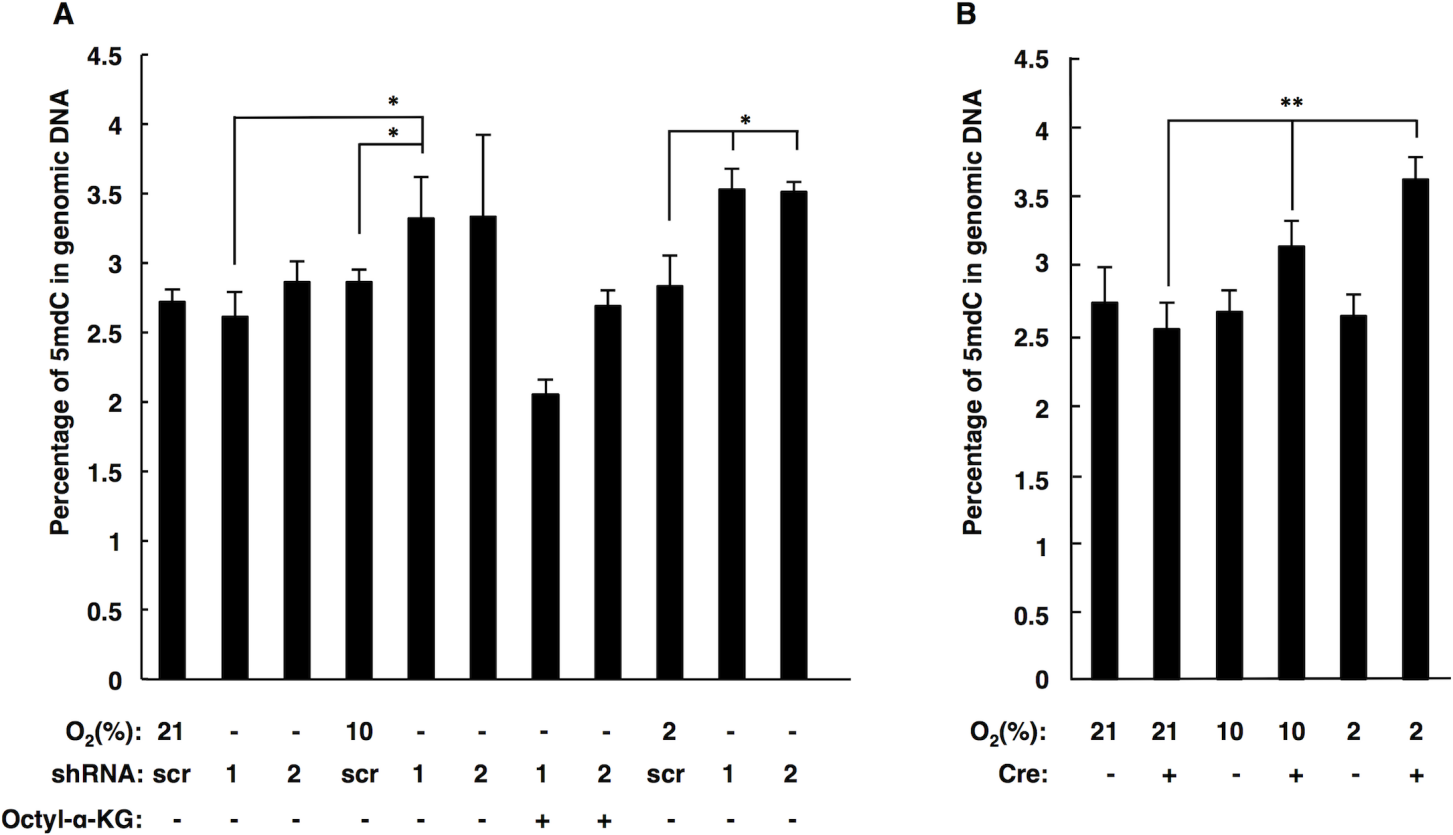
Oxygen dependence of SDHB knockdown and SDHC knockout on cytosine methylation in genomic DNA. A. 5-methylcytosine levels by HPLC-MS for SDHB knockdown cells exposed to different oxygen concentrations. Data are representative of at least three independent experiments. B. 5-methylcytosine levels by HPLC-MS for SDHC knockout iMEFs exposed to different oxygen concentration. Data (mean ± standard deviation) are representative of at least three independent experiments. Statistical significance by T-test (*P<0.05 and **P<0.01) is indicated.

Since succinate competes with α-KG at the active site of α-KG-dependent dioxygenases, we and others have shown that increasing α-KG concentration suppresses succinate inhibition of α-KG-dependent dioxygenases. [10,14]. α-KG has also been shown to overcome hypoxic inactivation of PHD [54]. To confirm this in our SDH loss cell culture models grown in 10% oxygen, we examined effects of α-KG addition. Two cell-permeable α-KG esters, dimethyl-α-KG and octyl-α-KG, were assessed. Western blot analysis indicated that octyl-α-KG was a superior compound for this experiment because dimethyl-α-KG did not suppress the effects of succinate accumulation nor reverse the effects of hypoxia on PHD. In contrast, octyl-α-KG readily reversed the effects of hypoxia and succinate inhibition in hypoxic HEK293 cells, knockdown and knockout SDH loss models (S6 Fig, Fig 2C, 2D and Fig 3A). The basis for this difference in the behavior of α-KG analogs is unknown. However the analogs carry different charges, and the rates of membrane diffusion and cytoplasmic de-esterification are unknown. In addition, dimethyl-α-KG was considerably more toxic to cultured cells, complicating interpretation of its effects.

### TET inhibition by succinate is oxygen dependent in SDH loss models of PGL

Members of the TET family of 5-methylcytosine (5mdC) hydroxylases are also α-KG-dependent dioxgenases that are inhibited by succinate accumulation upon SDH loss [11,12,55]. We investigated whether TET inhibition is oxygen-dependent in our SDH loss cell culture models. 5mdC levels measured by LC-MS analysis in cells suffering succinate accumulation showed a gradual increase with decreasing oxygen concentrations, as predicted (Fig 3A). Specifically, SDHB knockdown increased 5mdC from 2.7% to 3.5% of total dC when comparing 21% and 2% oxygen, respectively. This result implies that the DNA hypermethylation phenotype upon succinate accumulation is exacerbated by hypoxia, as expected from the intrinsic enzyme reaction mechanism of the TET dioxygenase. Succinate inhibition of TET activity in SDHB knockdown cells was reversible with octyl-α-KG treatment (Fig 3A). In agreement with previous reports, 5mdC levels did not change in control cell cultures between the three oxygen conditions [56].

We also subjected nucleosides from TAM-treated *Sdhc* ^*fl/-*^ cre+ and *Sdhc* ^*fl/-*^ cre- iMEFs to LC-MS analysis after culture from three oxygen conditions. Effects were similar to those observed for SDHB knockdown cells (Fig 3B). *Sdhc* ^*fl/-*^ cre+ iMEFs treated with TAM showed increased 5mdC levels of 2.6%, 3.1%, and 3.5% of total dC in 21%, 10%, and 2% oxygen, respectively.

### Human PGL tumors show histone methylation accumulation, and 5hmC depletion

Our *in vitro* studies manipulated oxygen concentrations in an attempt to explore physiologically-relevant conditions. The observation that dioxygenase inhibition by succinate accumulation is profoundly dependent on oxygen concentration raises the question whether hypoxia in SDH loss tumors is sufficient to drive similar inhibition. We therefore analyzed dioxygenase reaction products in three PGL tumors, one sporadic PGL, and control tissues by immunohistochemistry. The relevant clinicopathological characteristics of the PGL specimens are summarized in S1 Table, noting that PGL 7 and 8 were the most aggressive. Reported germline SDH mutations were confirmed for PGL5, PGL7, and PGL8 but not the sporadic (Spo.) PGL (S7 Fig). An isocitrate dehydrogenase (IDH) gain-of-function mutant glioma tumor specimen was used as a positive control because 2-hydroxyglutarate accumulation in such tumors reportedly inhibits α-KG-dependent dioxygenases [57–60]. Normal kidney and two specimens of normal abdominal ganglia served as negative controls. It has been reported that SDHB protein loss accompanies any disruption of the SDH complex in PGLs [43,44]. We therefore stained for SDHB, and found all PGL tumors to be negative, as expected (S8 Fig).

**Fig 4:**
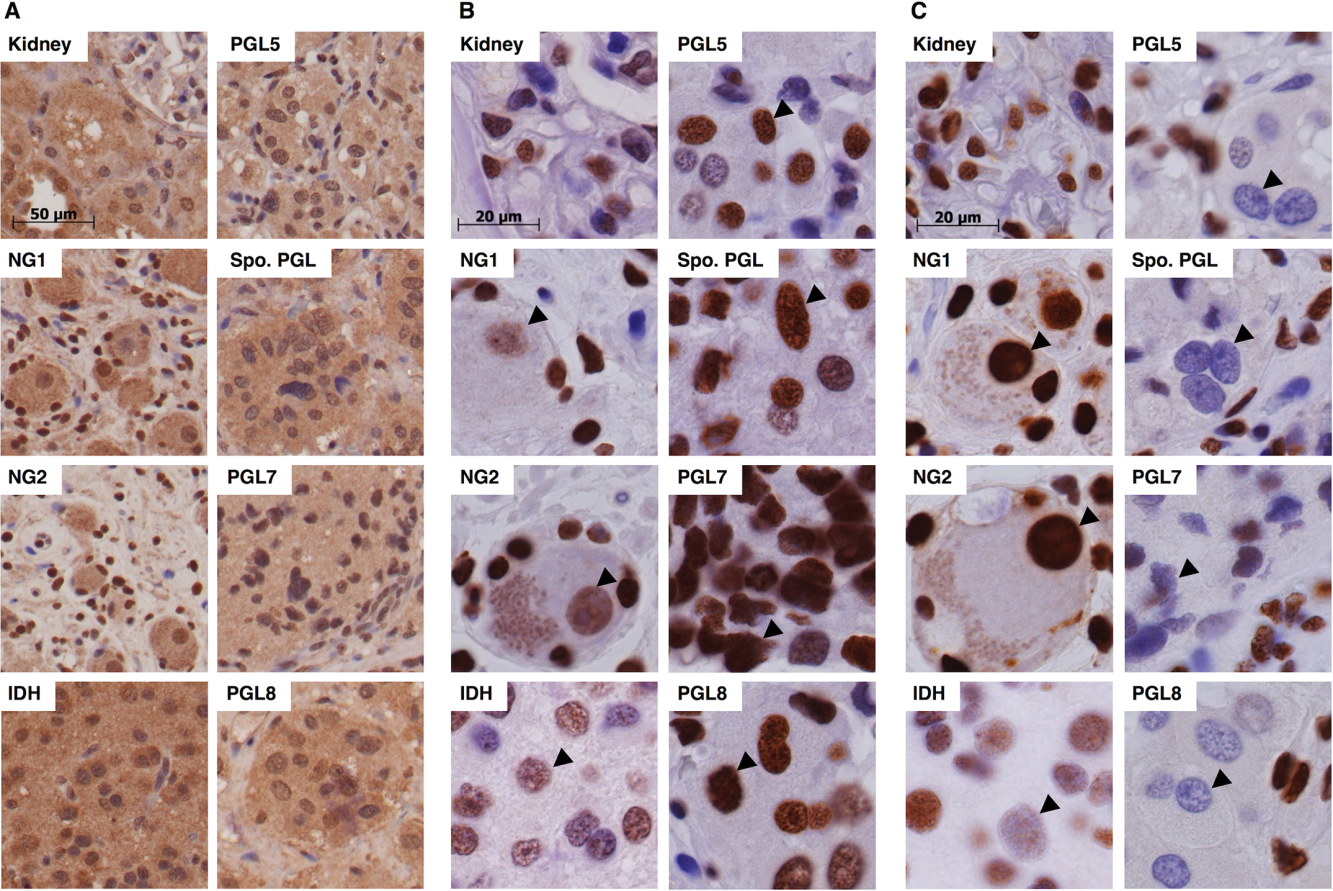
HIF1α and H3K9me2 accumulation and 5-hydroxy-methyl-2’-deoxycytidine (5hmdC) depletion in PGL specimens compared to controls. Normal ganglia 1 (NG1), normal ganglia 2 (NG2) and IDH-mutant (IDH). Sporadic PGL (Spo. PGL). A. HIF1α staining. B. HIF2α staining. C. H3K9me2 staining. Arrows indicate H3K9me2 staining in nuclei of neurons or chief cells. D. 5hmdC staining. Arrows indicate 5hmdC staining in the nuclei of neurons and chief cells.

To explore whether the combination of endogenous succinate accumulation and hypoxia was sufficient to drive dioxygenase inhibition in these PGL tumor specimens, we evaluated levels of HIF1α, HIF2α, histone methylation, and cytosine methylation. All are expected to increase upon dioxygenase inhibition [8]. All specimens showed weak diffuse cytoplasmic and nuclear staining for HIF1α. HIF1α staining in PGL was indistinguishable from the IDH positive control known to exemplify pseudohypoxia (Fig 4A). In contrast, HIF2α staining appeared to be lower in PGLs than both the IDH-mutant and normal ganglia tissues (Fig 4B). H3K9me2 was dramatically higher in PGL tumors compared to controls (Fig 4C). Antibodies specific for H3K9me2 stained chief cell nuclei of PGL cells. Comparison was made between these nuclei and nuclei of normal ganglia tissues (Fig 4C, arrows). Finally, inhibition of TET activity should lead to 5mdC accumulation and 5hmdC depletion. Indeed, 5hmdC was strongly depleted in PGL tumors (Fig 4D) with the extent of depletion correlated with reported tumor aggressiveness.

## Discussion

The fascinating kinetic reaction mechanism of α-KG-dependent dioxygenases has been well established [46,47]. The enzyme binds and splits molecular oxygen at an Fe (II)-dependent active site that also binds α-KG and the macromolecular substrate, in an ordered tri-tri reaction mechanism in which modified substrate, succinate and CO_2_ are products. This highly-exothermic reaction coupled with α-KG decarboxylation make the chemistry essentially irreversible. The driving force (free energy change) for the reaction depends on the equilibrium concentrations of the reactants and products because the reaction rates obey mass action relationships. Thus, the forward reaction rate is proportional to the concentrations of each of the three reacting substrates. Importantly for the present discussion, dioxygenase enzymes are subject to inhibition by hypoxia, by loss of the Fe (II) cofactor [61], or by inhibitors that compete with α-KG, such as succinate (itself a reaction product) or 2-hydroxyglutarate [9,11,45,58,60,62–64]. The work described here demonstrates that the function of PHD, Jumonji domain, and TET dioxygenases in living cells can be controlled by manipulating the relative concentrations of reactants (α-KG and oxygen) and the competitive inhibitor, succinate. In agreement with reports that succinate accumulates in SDH loss tumors [15,16], we confirm succinate accumulation in our SDH loss cell models, and show that the succinate:α-KG ratio (which determines the extent of dioxygenase inhibition) is dramatically increased upon SDH loss. Importantly, we confirm that dioxygenase poisoning by succinate can be suppressed by increasing α-KG concentration *or increasing oxygen*.

It is interesting to compare our results with previous reports. We find that hypoxic culture conditions are required for succinate inhibition of dioxygenases. Room air (21% oxygen, actually a *hyperoxic* condition relative to tissue) did not support dioxygenase inhibition in our models. Closer examination revealed various differences from previous studies, including the method of SDH knockdown, the cell types studied, and the concentration of DMS employed. HIF1α elevation was reported upon 2.5 mM and 20 mM DMS treatment of HEK293 cells in normoxia [9,11]. In agreement with a previous report [45], we could not replicate this experiment with 20 mM treatment of DMS in 21% oxygen. Possible experimental differences include the knockdown strategy. We employed a lentiviral shRNA stable knockdown approach that allowed greater than 90% knockdown of SDHB for at least 10 passages. This approach is different from the siRNA strategy employed by Selak et al., MacKenzie et al., and Xiao et al. Though these alternative knockdown strategies have similar functional outcomes, they differ in their molecular mechanisms of action, affected RNA metabolism pathways, kinetics, and various other factors [65,66]. Among these differences are cell processing and handling times, which could conceivably play a role in the different results. In the case of SDH conditional knockout studies, differences in cell type may explain the different oxygen effects. Mouse chromaffin cells were used in the previous report [12]. We studied total kidney cells and MEFs.

Both HIF1α and HIF2α signaling have been proposed to be important in PGL tumorigenesis [67–69], though the relative contributions are unknown. According to the literature, both proteins are overexpressed in SDH-deficient cell culture models [9] and in some PGL specimens [8,17,20]. There is some evidence that HIF2α plays a larger role in PGL tumorigenesis. HIF2α reportedly contributes to an immature chromaffin cell phenotype [70]. HIF2α was reportedly expressed at a higher level in SDH loss PGLs than in sporadic PGLs [18,19], and at higher levels than HIF1α in SDH loss PGLs [20]. As expected, the cell culture models we report here confirm stabilization of both HIF1α and HIF2α upon SDH loss, and we clarify that this result depends on the degree of hypoxia (Fig 2). In addition, DNA and histone hypermethylation were observed in all models as a function of hypoxia, and also in PGL tumors (Figs 2–4). Interestingly, the human PGL specimens we report display HIF1α levels comparable to normal control tissues (Fig 4A) and HIF2α staining is lower than in normal ganglia and IDH-mutant controls (Fig 4B). According to these results, epigenetic changes secondary to DNA and histone hypermethylation may play dominant roles in growing tumors relative to HIF signaling. This result does not clarify whether HIF1α and/or HIF2α stabilization may drive the initial process of PGL tumorigenesis.

The results of this study are significant for the understanding and potential treatment of familial PGL. If the succinate accumulation hypothesis [9,71] is indeed correct, the underlying molecular pathology of SDH-loss PGL tumors is genome-wide epigenetic reprogramming related to aberrant stabilization of HIFα and HIF2α transcription factors, hypermethylation of histones and hypermethylation of DNA [11,12]. How this epigenetic reprogramming selectively transforms neuroendocrine cells is unknown. However, the poisoning of dioxygenase enzymes secondary to succinate accumulation becomes a potential target for understanding PGL epidemiology and novel therapeutics.

From the perspective of epidemiology, the observation that PGL incidence has a peculiar correlation with reduced environmental oxygen (altitude effects) and chronic hypoxia in patients with respiratory or circulatory pathologies [23,24,30,72–74] is strikingly explained by the molecular effects of hypoxia that we illustrate here. While patients suffering from circulatory or respiratory failure are chronically hypoxic, the effects of altitude are more complex. Oxygen homeostasis through increased blood hemoglobin and other physiological responses might be expected to rapidly compensate for hypoxia due to altitude. If such compensation is complete, tissues of patients at altitude should be no more hypoxic than at sea level. However, if compensation is incomplete [75], it is plausible that hypoxia synergizes with succinate accumulation to increase PGL prevalence and severity via the fundamental dioxygenase enzyme reaction mechanism shown here.

From the perspective of therapies for SDH-loss familial PGL, the present results emphasize the potential for novel approaches in this malignancy. First, if appropriate animal models can be developed it may be possible to determine whether restoring SDH function (perhaps through viral transduction of a rescuing cDNA) is sufficient to reverse tumor growth. Second, and as been previously shown by us and others, succinate poisoning of dioxygenases can be overcome by increasing α-KG [10–12,14], raising the possibility of metabolite therapy. Third, SDH loss drives PGL cells to a state of aerobic glycolysis, suggesting that PGL cells will be uniquely sensitive to inhibitors of glycolysis [31]. Finally, the work reported here illustrates the potential of therapeutic hyperoxia in suppressing effects of succinate accumulation upon SDH loss. We show that raising oxygen tension in SDH loss cells from 10% to 21% is sufficient to restore dioxygenase function to normal levels. With the future development of SDH-loss animal models of familial PGL, it should be possible to test experimentally this intriguing therapeutic prediction by using supplemental oxygen [76].

## Supporting Information

S1 Fig Normal functions of Fe/O_2_/α-KG dependent dioxygenases in (A) HIF1α degradation, (B) histone demethylation, and (C) 5-methyldeoxycytosine hydroxylation (R, deoxyribose). It is hypothesized the SDH-mutant PGL cells experience succinate accumulation with succinate serving as a competitive inhibitor with α-KG at the dioxygenase active site. Dioxygenase reaction rate is predicted to be proportional to the concentrations of oxygen, substrate, and α-KG. Here we test the oxygen-dependence of succinate inhibition.
(TIFF)

S2 Fig Generation of Sdhc conditional knockout mice. Founder mice have a wild type (WT) SDHC allele and an *Sdhc* gene trap allele (-) in which an *Engrailed* polyadenylation site (En2 SA) terminates transcription creating a truncated mRNA. The *Sdhc* floxed allele (fl) mice was created by FLP recombination between *FRT* sites (diamonds) in a prior breeding, yielding loxP recombination sites flanking *Sdhc* exon 4 in the fl allele. Mating between *Sdhc* (-/WT) and *Sdhc* (fl/WT) mice yielded *Sdhc* (-/fl) mice. Breeding onto a CRE^ER^-TM background allows disruption of both *Sdhc* fl alleles by recombination between loxP sites (triangles) upon Tamoxifen (TAM) treatment. Genotyping primers are indicated by arrows.
(TIFF)

S3 Fig Characterization of *Sdhc* knockout primary kidney cells model of PGL. A. PCR genotyping with primers reporting floxed (fl) and recombined *Sdhc* knockout alleles. B. Western blot of total lysate from primary kidney cells treated with TAM for 7 d. β-actin was used as a loading control. C. SDH enzyme activity in mitochondria isolated from primary mouse kidney cells with or without Cre-recombinase expression, treated with ethanol (EtOH) or TAM for 7 d. D. Relative metabolite levels in the indicated whole cell lysates.
(TIFF)

S4 Fig Effect of cell permeable dimethyl succinate (DMS: panel A) on histone methylation (B) and HIF1α accumulation (C) in the presence of 10% O_2_ or 21% O_2_. HEK293 cells were treated with 20 mM DMS and incubated in either 10% O_2_ or 21% O_2_ for 12 h prior to harvesting for Western blot analysis with anti-HIF1α, anti-H3K27me2, or anti-H3K9me2. Actin and total H3 were used as loading controls.
(TIFF)

S5 Fig HIFα accumulation and histone hypermethylation in primary *Sdhc* knockout kidney cells as a function of oxygen concentration. Cells were treated with 1 μM tamoxifen for 7 d prior to analysis.
(TIFF)

S6 Fig Effects on dioxygenase function of dimethyl-α-KG (A) and octyl-α-KG (B) in the presence of succinate accumulation. (TIFF)

S7 Fig PGL DNA mutation analysis by sequencing PGL5, Sporadic PGL (Spo. PGL), PGL7, and PGL8 tumor DNA. Spo. PGL has been confirmed to be a paraganglioma without detected known mutation.
(TIFF)

S8 Fig Characterization of PGL tumor staining for SDHB. (TIFF)

S1 Table Human PGL tumor specimen origins and clinicopathology characteristics. (TIFF)
