# Supplementary figures and images for: Oxygen Concentration Controls Epigenetic Effects in Models of Familial Paraganglioma
Source: PLoS One. 2015 May 18;10(5):e0127471. doi: 10.1371/journal.pone.0127471 (PMC4436181; doi:10.1371/journal.pone.0127471)

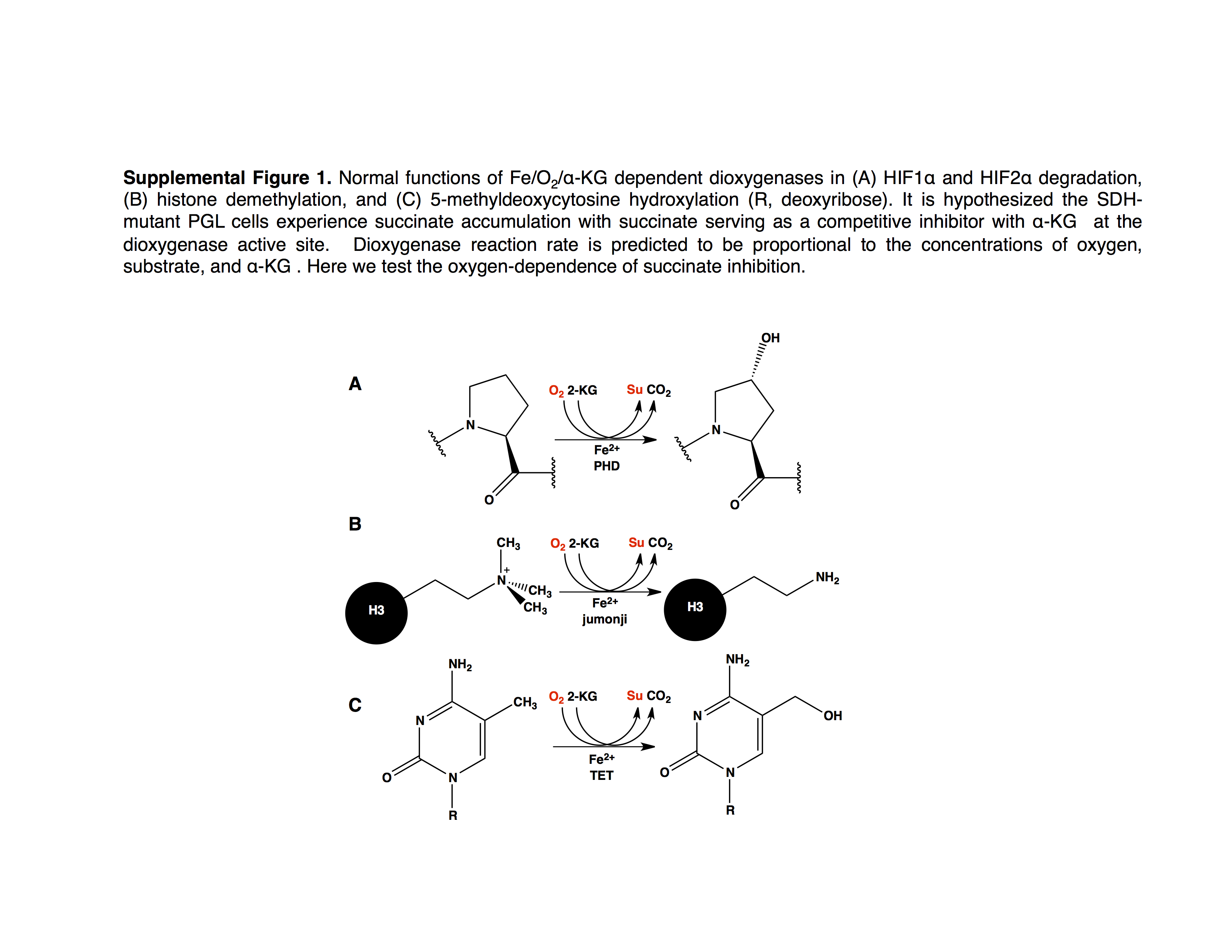

Supplement: S1 Fig — It is hypothesized the SDH-mutant PGL cells experience succinate accumulation with succinate serving as a competitive inhibitor with α-KG at the dioxygenase active site. Dioxygenase reaction rate is predicted to be proportional to the concentrations of oxygen, substrate, and α-KG. Here we test the oxygen-dependence of succinate inhibition. (TIFF) [file pone.0127471.s001.tiff]

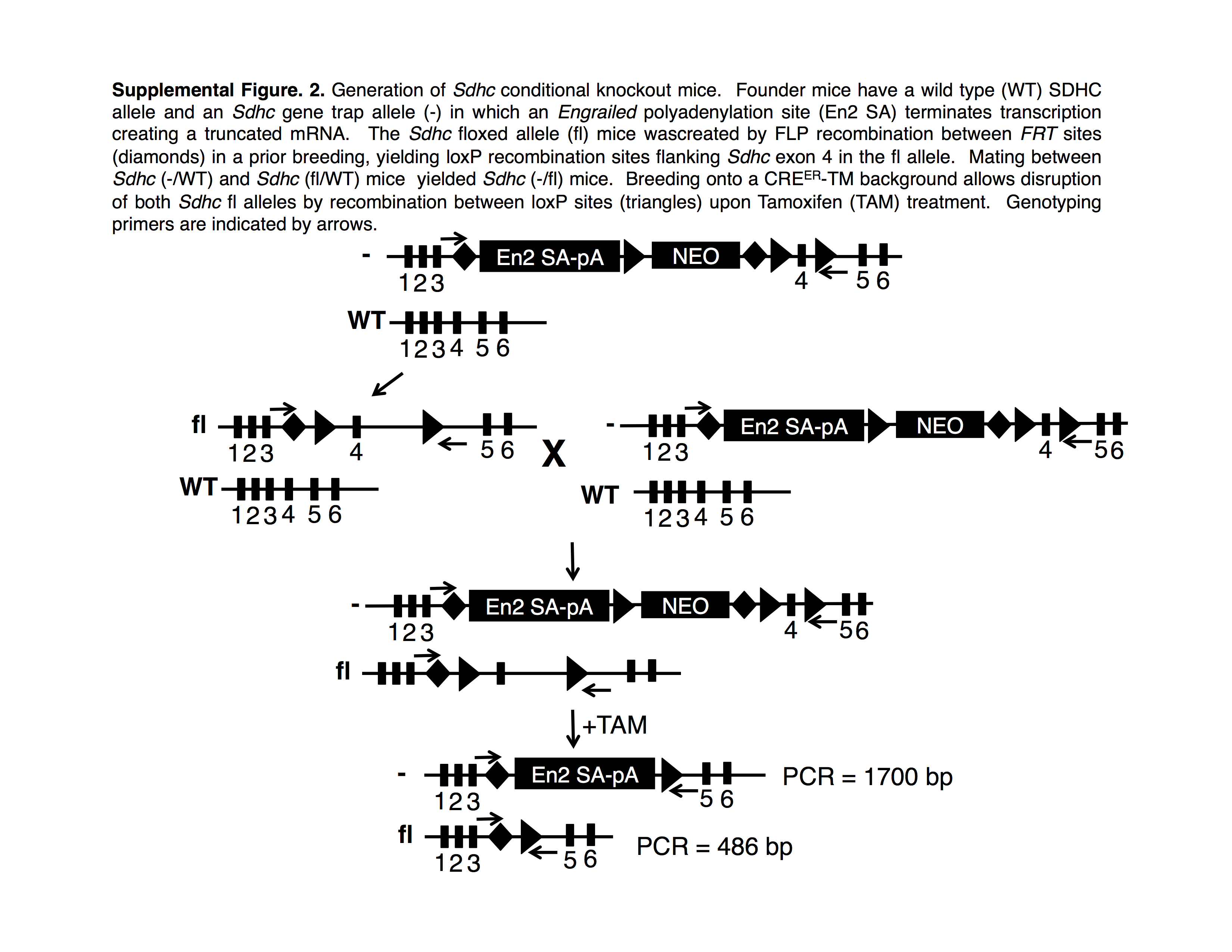

Supplement: S2 Fig — Founder mice have a wild type (WT) SDHC allele and an Sdhc gene trap allele (-) in which an Engrailed polyadenylation site (En2 SA) terminates transcription creating a truncated mRNA. The Sdhc floxed allele (fl) mice was created by FLP recombination between FRT sites (diamonds) in a prior breeding, yielding loxP recombination sites flanking Sdhc exon 4 in the fl allele. Mating between Sdhc (-/WT) and Sdhc (fl/WT) mice yielded Sdhc (-/fl) mice. Breeding onto a CREER-TM background allows disruption of both Sdhc fl alleles by recombination between loxP sites (triangles) upon Tamoxifen (TAM) treatment. Genotyping primers are indicated by arrows. (TIFF) [file pone.0127471.s002.tiff]

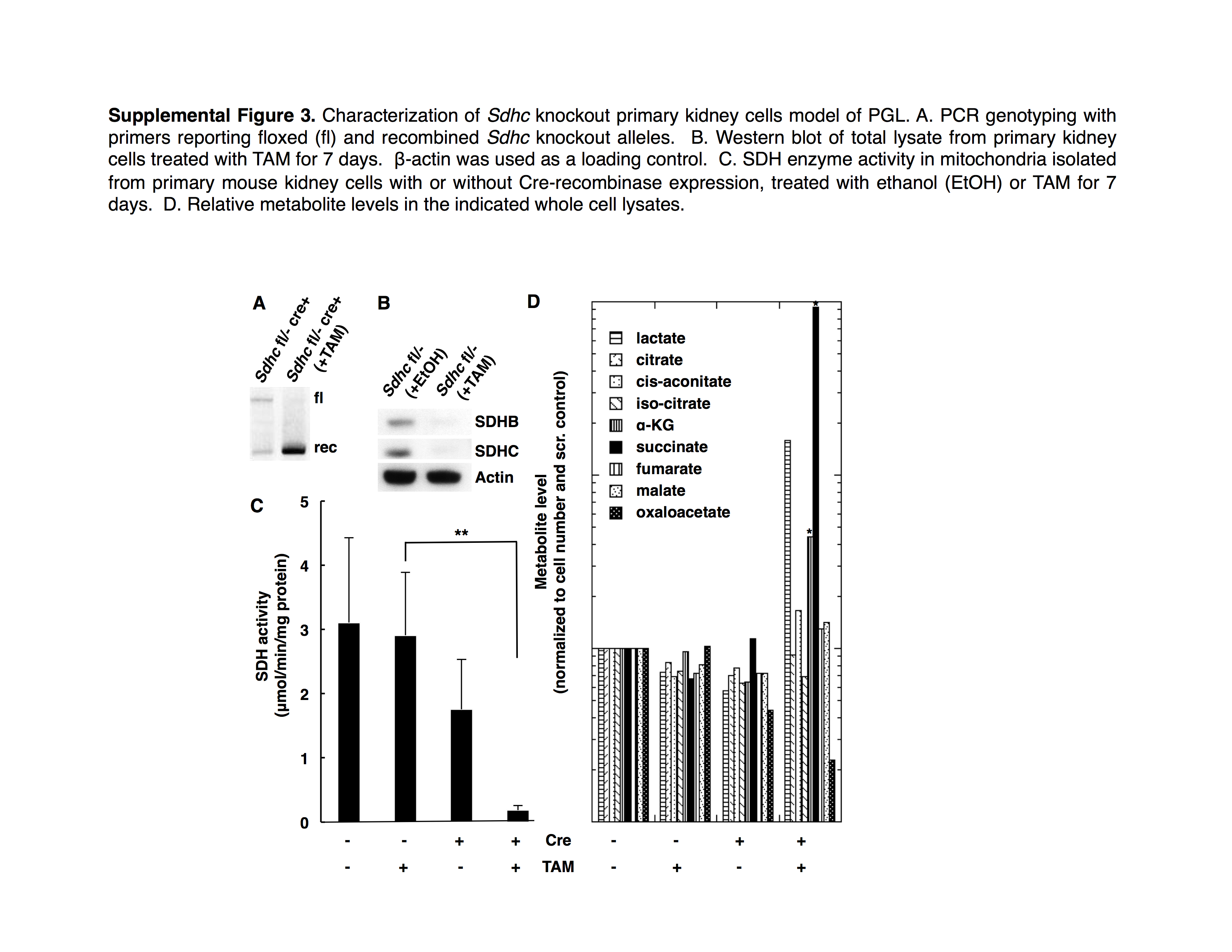

Supplement: S3 Fig — A. PCR genotyping with primers reporting floxed (fl) and recombined Sdhc knockout alleles. B. Western blot of total lysate from primary kidney cells treated with TAM for 7 d. β-actin was used as a loading control. C. SDH enzyme activity in mitochondria isolated from primary mouse kidney cells with or without Cre-recombinase expression, treated with ethanol (EtOH) or TAM for 7 d. D. Relative metabolite levels in the indicated whole cell lysates. (TIFF) [file pone.0127471.s003.tiff]

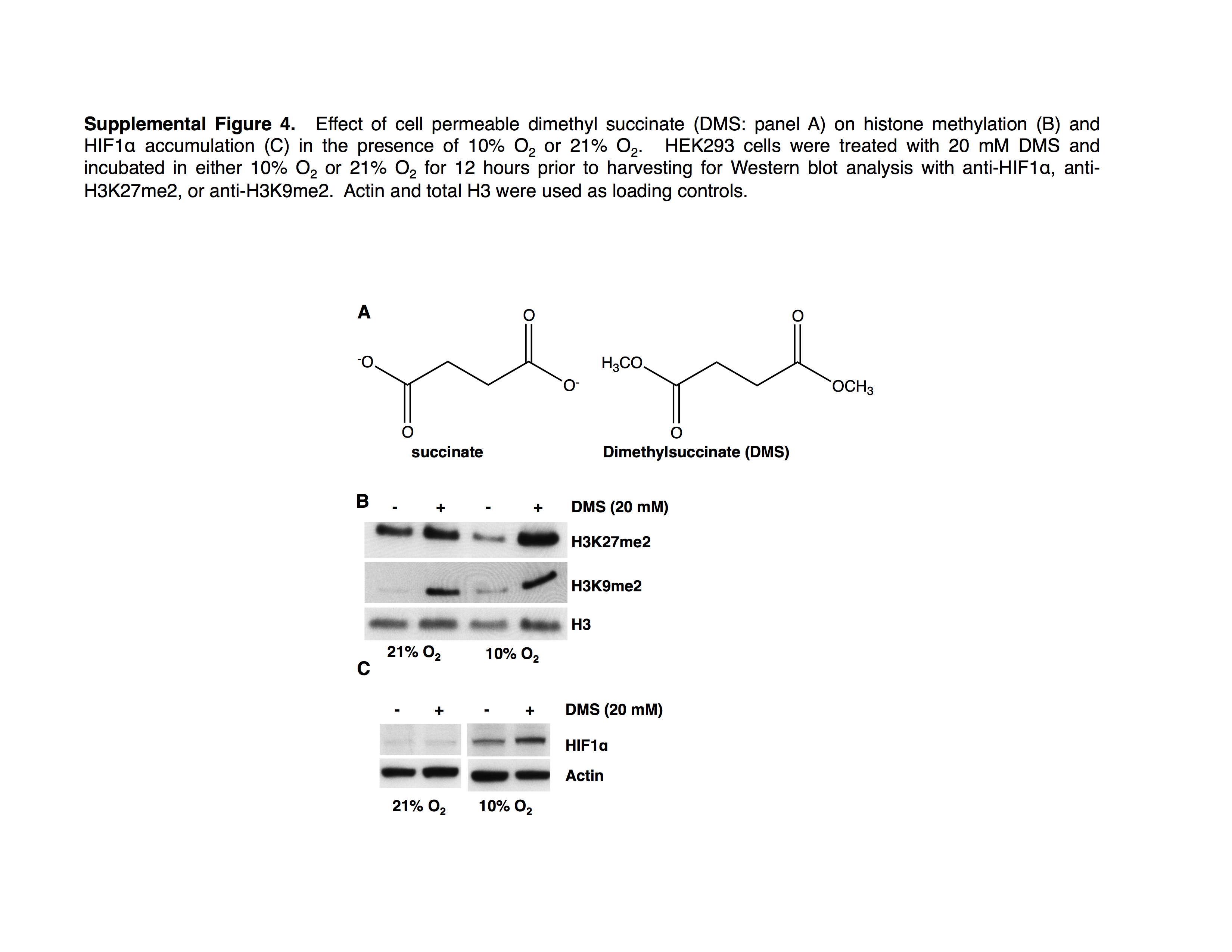

Supplement: S4 Fig — HEK293 cells were treated with 20 mM DMS and incubated in either 10% O2 or 21% O2 for 12 h prior to harvesting for Western blot analysis with anti-HIF1α, anti-H3K27me2, or anti-H3K9me2. Actin and total H3 were used as loading controls. (TIFF) [file pone.0127471.s004.tiff]

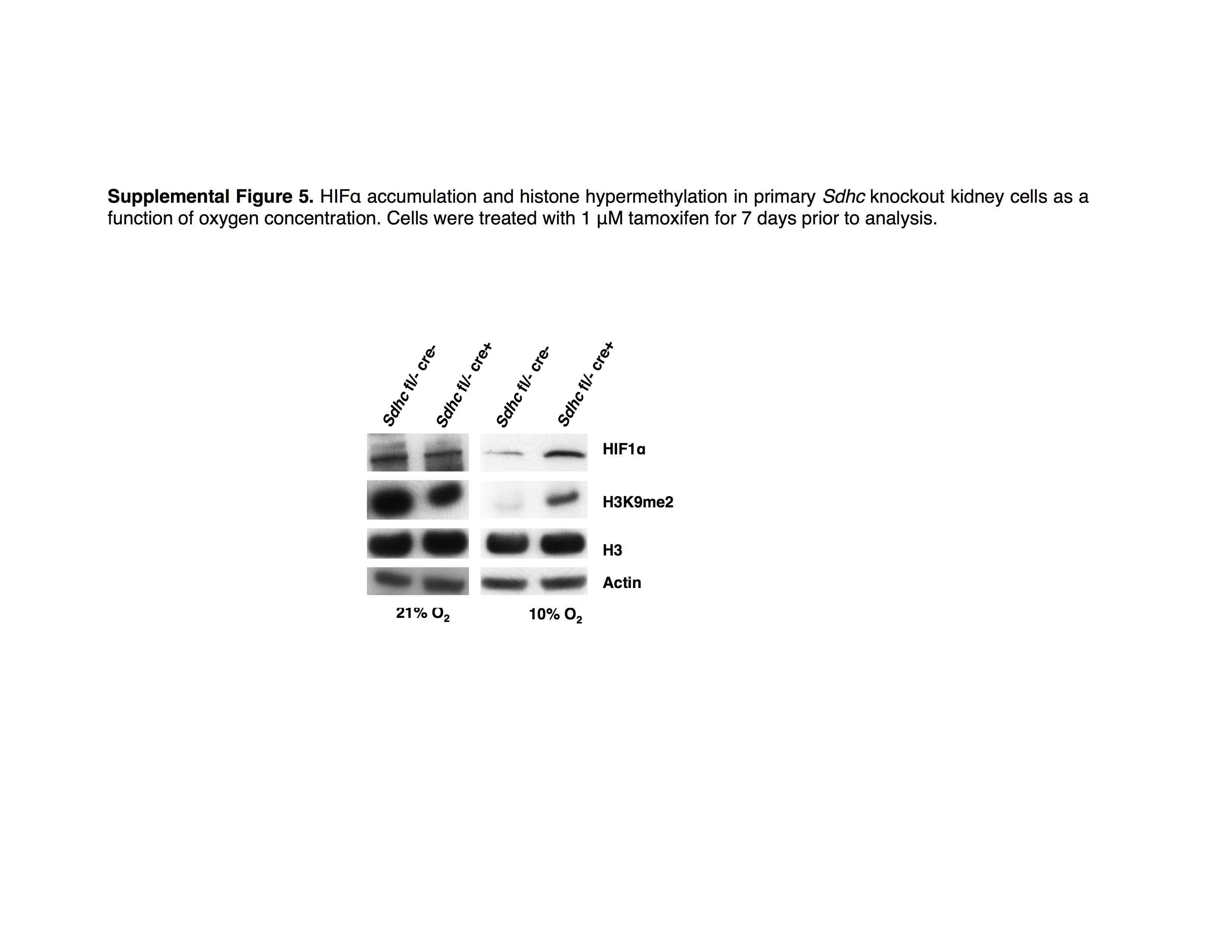

Supplement: S5 Fig — Cells were treated with 1 μM tamoxifen for 7 d prior to analysis. (TIFF) [file pone.0127471.s005.tiff]

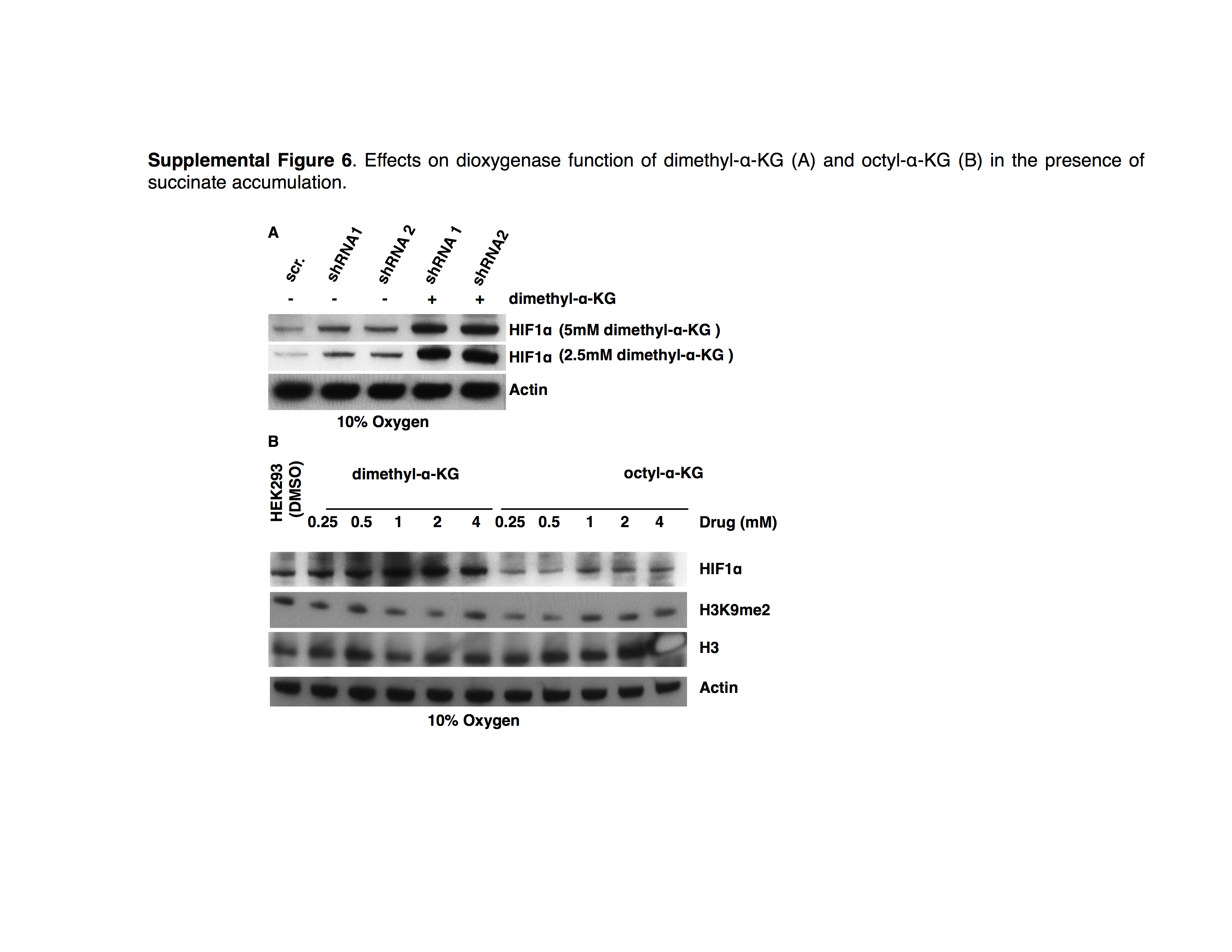

Supplement: S6 Fig — (TIFF) [file pone.0127471.s006.tiff]

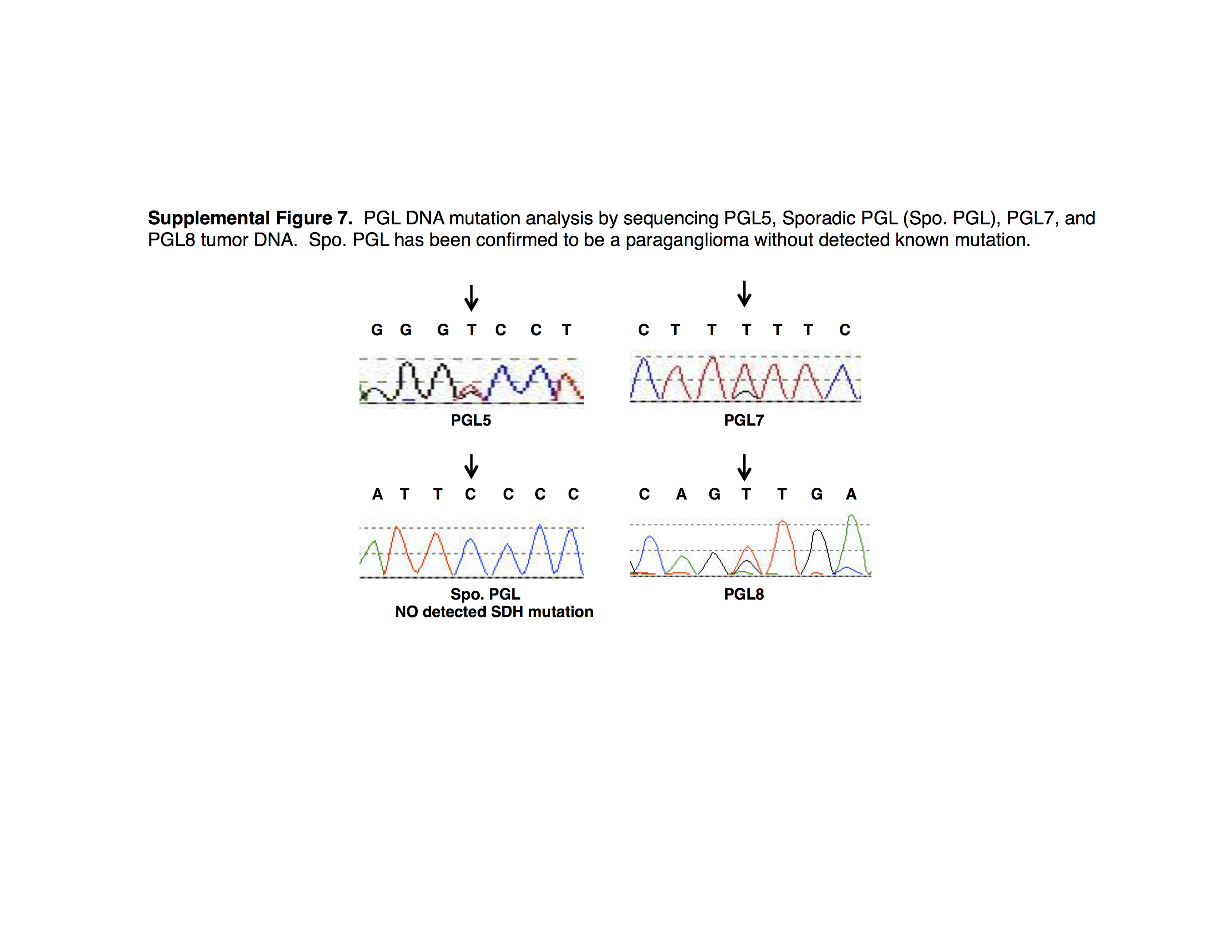

Supplement: S7 Fig — Spo. PGL has been confirmed to be a paraganglioma without detected known mutation. (TIFF) [file pone.0127471.s007.tiff]

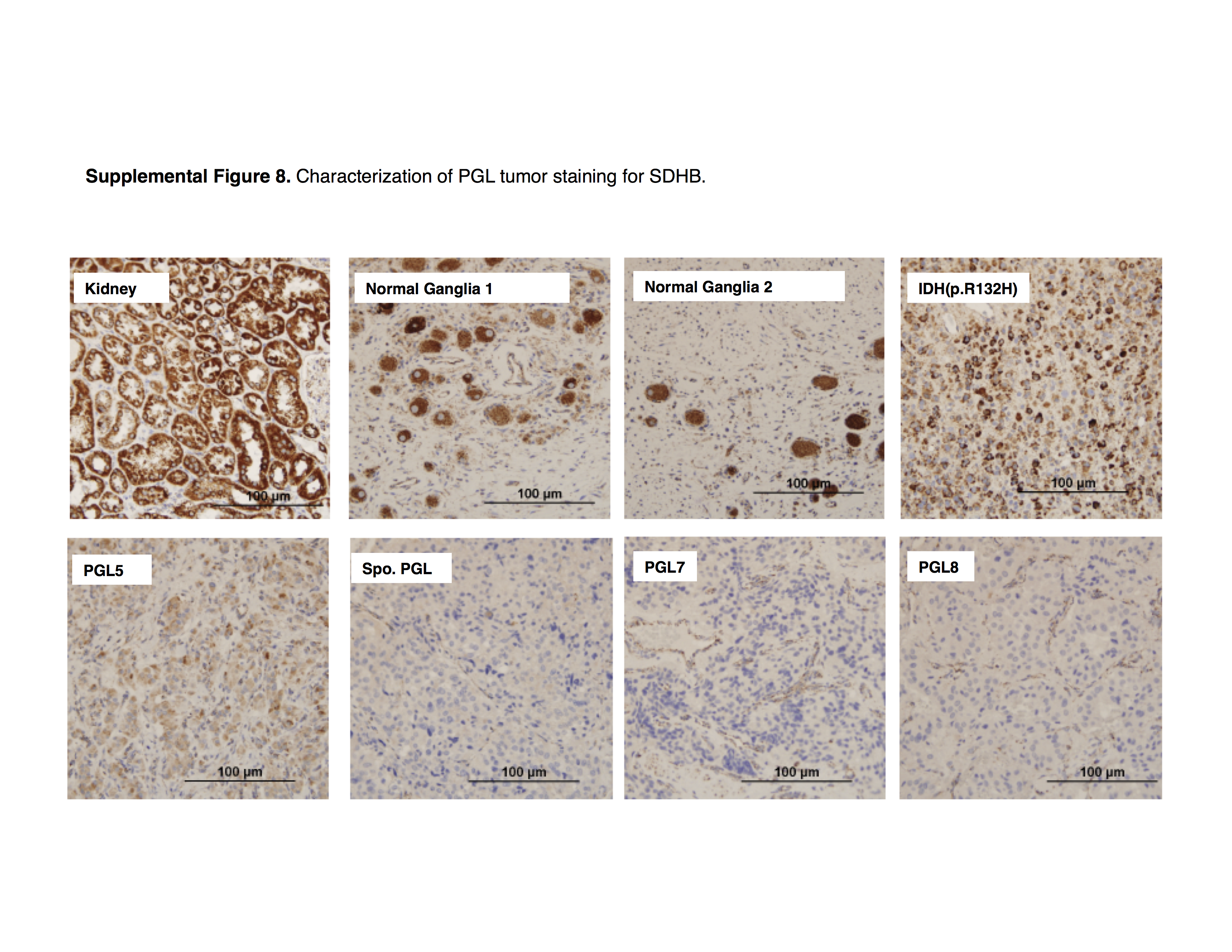

Supplement: S8 Fig — (TIFF) [file pone.0127471.s008.tiff]

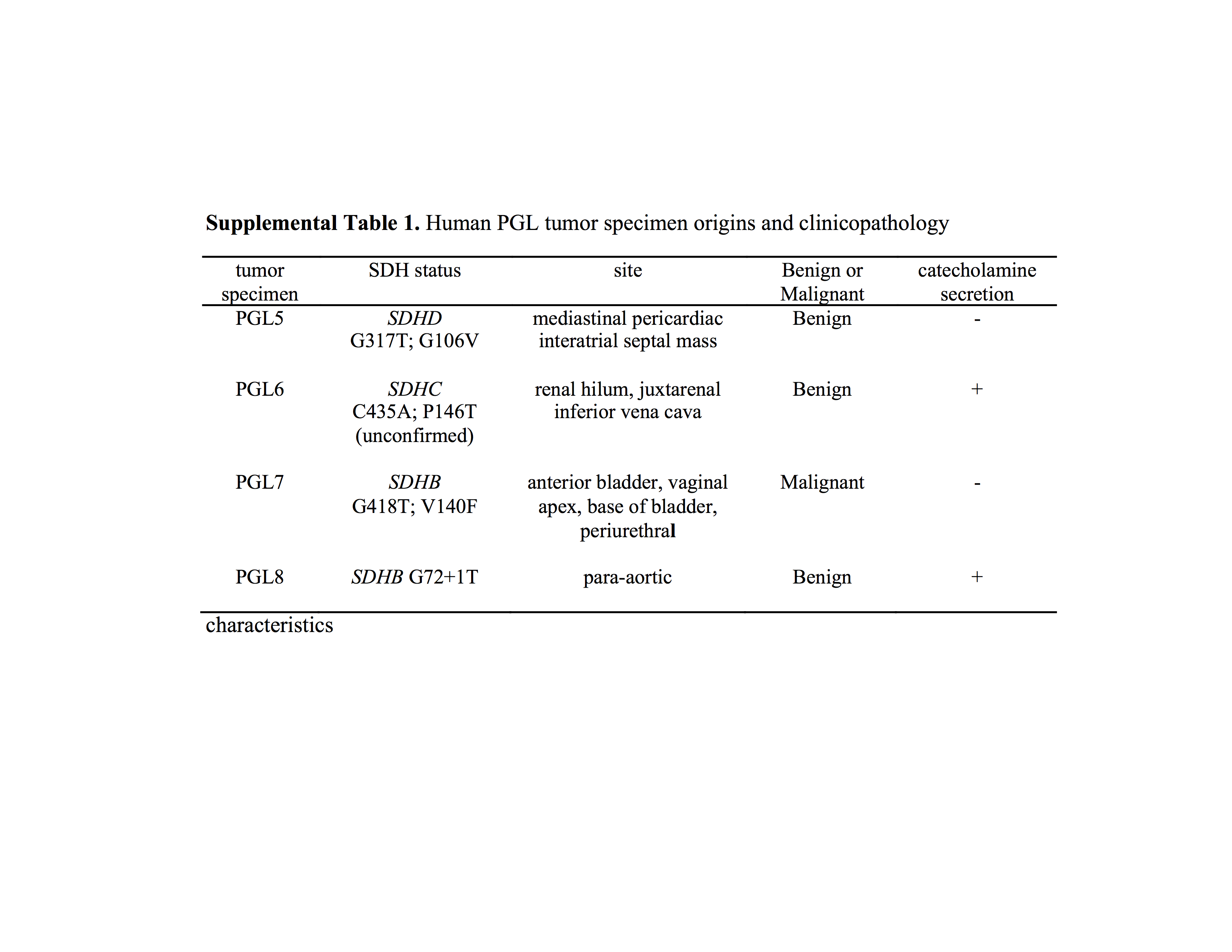

Supplement: S1 Table — (TIFF) [file pone.0127471.s009.tiff]
